# Allergies to Titanium Dental Implants: What Do We Really Know about Them? A Scoping Review

**DOI:** 10.3390/biology9110404

**Published:** 2020-11-18

**Authors:** Rubén Comino-Garayoa, Jorge Cortés-Bretón Brinkmann, Jesús Peláez, Carlos López-Suárez, Jose María Martínez-González, María Jesús Suárez

**Affiliations:** 1 Department of Conservative Dentistry and Orofacial Prosthodontics, Faculty of Dentistry, Complutense University of Madrid, 28001 Madrid, Spain; comino93garayoa@gmail.com (R.C.-G.); jpelaezr@ucm.es (J.P.); carlopezsuarez@gmail.com (C.L.-S.); mjsuarez@odon.ucm.es (M.J.S.); 2 Department of Dental Clinical Specialties, Faculty of Dentistry, Complutense University of Madrid, 28001 Madrid, Spain; jmargo@odon.ucm.es

**Keywords:** titanium implants, corrosion, hypersensitivity, allergic reactions, scoping review

## Abstract

**Simple Summary:**

The scientific literature repeatedly insists on the success of titanium implants. Nevertheless, the so-called tribocorrosion process releases titanium ions into the surrounding tissues, which can trigger a cascade of reactions, localized or at a distance, or even systemic reactions. Consequently, guidelines should be drawn up before starting treatment; when a hypersensitivity reaction following titanium dental implant placement occurs, a range of treatment alternatives should be clearly established and made available.

**Abstract:**

The purpose of this scoping review was to describe the current state of knowledge and understanding of allergies to titanium dental implants. A scoping review was conducted following the Prisma Extension for Scoping Reviews checklist. An electronic search was performed in five databases complemented by manual and grey literature searches. Fifty-two relevant papers were included for final review. Titanium particles can be released from the surfaces of dental implants in a process called tribocorrosion, which may contribute to bone loss due to inflammatory reaction. Diverse mechanisms have been described that may trigger allergy to titanium, as well as the clinical signs that manifest as the allergy develops. Allergies to titanium are uncommon but represent a real possibility that should not be overlooked in patients requiring prosthodontic rehabilitation with dental implants. Allergy can trigger a range of symptoms. Patients who have already been diagnosed with allergies to other metals will be more predisposed to suffering an allergy to titanium. Further investigation is needed in order to measure the true scope of these allergies.

## 1. Introduction

Titanium (Ti) is a transition metal known for its high resistance to flexion and corrosion. Since the second half of the twentieth century, it has been used in many different fields: for military purposes and in aerospace, for sports equipment, jewelry, etc. [1,2,3]. In the field of medicine, it is used to fabricate pacemakers, endoprostheses, and stents. From the earliest days of implant dentistry, titanium has been considered the gold standard material for fabricating dental implants due to its excellent biocompatibility, strength, and osteointegration capacity [4], the latter being a key requirement for long-term implant stability. Furthermore, research has demonstrated that titanium implants offer a success rate in the range of 92.5% to 96.4% and a survival rate of 94.7% to 99.4% over observation periods of at least five years [5,6,7].

Most researchers agree that titanium is the least allergic metal among the materials of choice for biological purposes [8,9,10,11]. Nevertheless, the literature includes reports of isolated cases of hypersensitivity related to titanium and/or the chemical components of titanium alloys, a situation that necessitates further investigation.

Since the first case report describing a pacemaker in 1984 [12], various cases of titanium allergies have been published [8,13]. The first case involving dental implants was reported in 2008 [14]. The literature contains only a small number of studies investigating allergies to dental implants, and these seem to lack standardized protocols, firm inclusion criteria, and medium- or long-term follow-up periods. Moreover, most of the available literature consists of case reports.

This lack of information justifies the present scoping review, which set out to address the following aims in edentulous and partially edentulous patients rehabilitated by means of titanium dental implants: mechanisms that can trigger allergy to titanium, clinical manifestations derived from allergy to titanium, diagnostic tests to identify and prevent anomalous reactions to titanium and its chemical components, reported cases and clinical studies of allergic hypersensitivity to titanium dental implants, and therapeutic options available to deal with cases of allergy to titanium.

## 2. Materials and Methods

### 2.1. Search Strategy

The present scoping review followed the guidelines of Preferred Reporting Items for Systematic reviews or Meta-Analyses extension for Scoping Reviews (PRISMA-ScR) [15]. The practice-orientated research question was: What is the current state of knowledge regarding allergies to titanium dental implants?

A comprehensive literature search covered a period from the first published case to 22 April 2020, using five electronic databases: MEDLINE/PubMed, Embase, Scopus, Web of Science and the Cochrane Library. In addition, manual and gray literature searches were conducted. The principal key search terms employed, alone or in combination with Boolean operators, for the different searches were as follows: “allergy” or “hypersensitivity” and “dental implants.” This search strategy was adapted for use in the various databases. 

### 2.2. Eligibilty Criteria

The inclusion criteria were: Articles that review hypersensitivity to titanium, related mechanism of action, clinical manifestations, diagnostic test, cases of hypersensitivity to titanium dental implants and subsequent management. Articles published in English, Spanish, and German were included. Both in vivo and in vitro studies were considered. Given the scarce literature available, isolated case reports and case series were included in this review.

No exclusion criteria were applied, excepting the language.

The titles and abstracts identified in the initial search were assessed by two authors (R.C.-G. and J.C.-B.B.) for eligibility after eliminating duplicated articles. The studies that appeared to meet the inclusion criteria were retrieved in their full-text version and assessed. A manual search for additional relevant titles was also carried out in each article’s bibliography section. Any disagreement between these two reviewers was resolved by discussion with all the authors until consensus was reached.

### 2.3. Data Extraction and Collection

Having identified articles that met the inclusion criteria, the following data were extracted using a pre-piloted specific form: First author’s name; publication year; country; research design; sample size (in cases of cohort, or case-control studies); and the authors’ main reported outcomes, findings, and recommendations. This information was then synthesized in different sections. These tasks were performed by the same two authors (R.C.-G. and J.C.-B.B.).

## 3. Results

**Figure 1:**
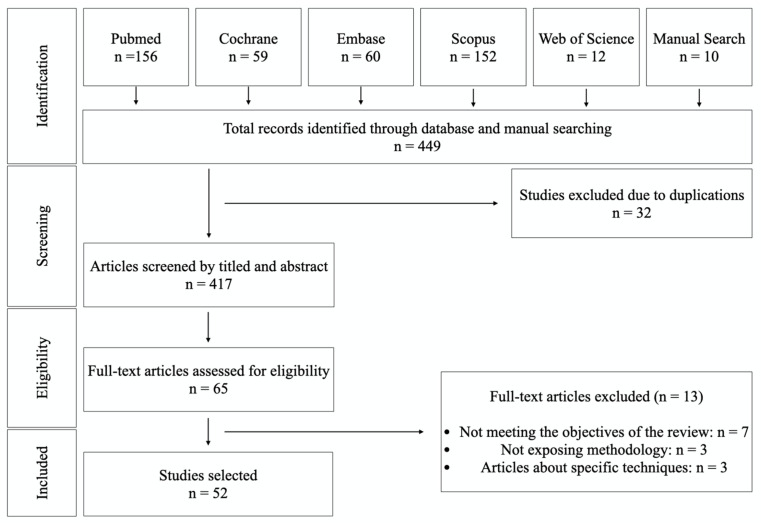
Flow chart of the inclusion and exclusion of studies in this review.

The initial search yielded 434 references and 10 were identified in the manual search (*n* = 449). After removing duplications, screening and applying inclusion criteria, 65 articles were included for data extraction and full-text assessment. However, 13 articles were excluded because they did not meet the objectives of the review, didn’t present a clear methodology, or were articles on specific techniques. Having read the full texts, 52 English language papers were selected as relevant to the objectives of the review. The PRISMA flowchart in Figure 1 summarizes the screening and selection processes. The selected articles were published between 1981 and 2020.

After exploring the final selection of studies, a large amount of relevant clinical information was condensed. The articles included 27 review articles, 11 clinical studies, 6 in-vitro studies, 3 in-vivo experimentations, and 5 case-reports. 

Of the 52 articles included in this review, seven papers deal exclusively with allergies to titanium dental implants, while the other included papers provide essential information for the understanding of this topic. The main outcomes of these reports are summarized in Table 1.

The articles came from 18 countries, spanning North America, Central and Eastern Asia, Europe, and South Africa.

### 3.1. Mechanisms that Can Trigger Allergies to Titanium

Allergic reaction to metal (in general and titanium in particular) follows the presence of ions deriving from implant corrosion, which may be ingested or come into contact with the skin or mucosa. These ions, although not sensitizing in themselves, can form complexes in combination with native proteins and act as allergens causing hypersensitivity reactions. The release of particles into the surrounding area, their later biodistribution in the body, and their final destination constitute a problem that lies at the center of research into biocompatibility and biokinetics [21].

#### 3.1.1. In Vitro

Titanium and its alloys show the highest resistance to corrosion of all metals. Commercially pure titanium forms a passive oxide surface film when exposed to an aqueous medium or air, which creates high immunity to corrosion by acids, chlorides, and wet environments, the degree of Ti ion elution being very small. This makes commercially pure titanium and its alloys virtually free of fretting corrosion, crevice corrosion, or pitting corrosion [22]. However, any break in the oxide layer can produce corrosion and affect biocompatibility [23].

In this context, it has been demonstrated that the passivating TiO2 layer is reformed in 0–200 ms after breakage on Ti-alloys, giving to these metals their outstanding corrosion resistance [24]. A low pH (<3) increases titanium corrosion, as does the absence of the oxide surface layer or crevice corrosion [9].

Titanium corrosion is of the electrochemical type and is produced in wet environments, in the presence of water, or other electrolytic fluids. This can lead to numerous corrosive species such as hydrogen ions, sulfur compounds, oxygen-free radicals, and chlorine ions, which can trigger alterations to the implant surface and peri-implant tissues; it is worth stressing that titanium corrosion potentially leads to these species mainly through the inflammatory response. The most common type of corrosion is galvanic, in which a breakage or displacement is produced in the titanium oxide surface layer.

Titanium has an innate resistance in aqueous chloride-containing environments [25]. When titanium is in a passive condition, due to the thin oxidation of titanium surfaces, corrosion rates are less than 0.02 mm/year [26] and well below the 0.13 mm/year, which is the maximum corrosion rate commonly accepted for biomaterial design and application [27].

However, once corrosion begins, it can develop into other types [28,29] such as crevice corrosion based on the propagation of ions inside the crevice, which in turn activates a host response that can be very rapid.

Another type of corrosion provokes tiny holes in the titanium (pitting corrosion); this happens when the metal’s anticorrosive potential is less than the corrosive potential of an attacking agent. However, this type of corrosion is very infrequent [27].

#### 3.1.2. In Vivo Experiments

- Non-dental implants

Stress during implant insertion can produce alterations, which trigger a process that exacerbates corrosion. It has been shown that titanium alloy in traumatology plates with a percentage of aluminum over 6% is more susceptible to this behavior. Corrosion will vary depending on the contaminants incorporated or the presence of manufacturing defects [30]. Moreover, Olmedo et al. conducted two animal experiments in which a TiO2 suspension was injected in rats, concluding that internal exposure will lead to a concentration of titanium ions in the surrounding tissues, lymph nodes, and even in lung or liver tissue [31,32].

The main outcomes of in-vivo experiments are summarized in Table 2.

- Dental implants

It has also been suggested that any mechanical disruption during insertion, or damage to the abutment connection, or the extraction of defective implants may cause particle release from the metallic structure. Suárez-López del Amo et al. [34] mention the higher prevalence of titanium particles of various sizes in peri-implant disease compared to healthy implants; in turn, the most common reasons for their release into the peri-implant medium being corrosion, implant insertion, implant-abutment friction, or wear derived from cleaning the implant. Concentrations of between 100 and 300 ppm have been reported in peri-implant tissues, often accompanied by discoloration [30].

Mombelli et al. [35] consider that there is some biological plausibility for a link between corrosion, presence of titanium particles, and biological complications. Mechanical wear and corrosion, together with environmental factors, contact to chemical agents, and interaction with substances produced by adherent biofilm and inflammatory cells, will lead in some cases to material degradation in a process called tribocorrosion. However, proof of a unidirectional sequence of causative events does not exist. These authors suggest that rather than being the trigger of disease, higher concentrations of titanium in peri-implantitis lesions could be the consequence of the presence of biofilms and inflammation.

The European Association for Osseointegration (EAO) Consensus Conference 2018 published a final statement [36], which includes an extensive discussion of the effects of titanium particles and biocorrosion on implant complications and subsequent survival rates. According to the statement, a number of in vitro studies have reported that the acidity of the oral environment caused by bacterial biofilm and/or inflammatory processes can provoke titanium particle release in a process known as “biocorrosion.” The resulting titanium debris upsets the balance between bone formation and resorption in two ways: through direct osteoclast and osteoblast activation and through the stimulation of inflammatory cytokine secretion from macrophages and lymphocytes.

Furthermore, a study using a sheep model and whose purpose was assessing the levels of dissemination of titanium from threaded screw-type implants following placement of single implants yielded inconclusive results. In this context only 2 implants failed to integrate, and these showed higher amounts of Ti in the lungs and regional lymph nodes compared with animals without failure. Notwithstanding, ambiguous results were found in the liver and spleen [33].

Titanium ions or titanium microparticles released into peri-implant tissues adjacent to an implant can trigger inflammatory reactions in the surrounding tissues. As said above, macrophages, activated by titanium, can secrete cytokines [19,36]. At the same time, due to their high affinity to proteins, titanium ions (haptens) combine with endogenous proteins to form antigenic molecules. These molecules are captured by Langerhans cells, related to T lymphocytes. In this way, sensitivity to titanium is characterized by the local presence of abundant macrophages and T lymphocytes and the absence of B lymphocytes [13,20,27,37].

Olmedo et al. [10] analyzed the peri-implant mucosa in 153 patients with submerged implants, finding that in 41% of cases, the mucosa presented metallic particles (metalosis) and T lymphocytes indicative of an immune response. According to Mine et al. [38], in addition to possible hypersensitivity reactions, titanium ions may inhibit osteoblast differentiation and alter both the percentage of RANKL (Receptor Activator for Nuclear Factor κ B Ligand) and osteoprotegerin responsible for osteoclast differentiation. In this way, titanium ions could have adverse effects on bone remodeling at the interface between implants and surrounding tissues [38,39].

The elements in titanium alloys can be classified as stabilized in the alpha phase, the beta phase, or both. Increasing the alpha phase boosts stability at high temperatures, while increasing the beta phase increases resistance to ambient temperature and the durability of medical titanium [3].

Titanium alloys (consisting mainly of titanium, aluminum, and vanadium: TiAl6V4) are the most widely used option for dental implants in comparison with pure titanium (TiO2), due to their higher strength. It should be noted that even ‘pure’ titanium has impurities that can trigger allergic reactions. These include traces of aluminum, beryllium, cadmium, iron, manganese, molybdenum, nickel, palladium, or vanadium [1,3,40,41,42,43].

Aluminum in implant alloys acts as an alpha phase stabilizer and reduces the weight of the alloy. Vanadium is a stabilizer of the beta phase and reduces the possibility of corrosion. More recently, vanadium-free alloys have been developed (Ti-6Al-7Nb and Ti-5Al-3Mo-4Zr) that exhibit equally good mechanical properties [3,30].

Various authors argue that aluminum could be related to persistent granulomas and recurring eczema, while beryllium can trigger allergies in the oral cavity mucosa [41,44].

### 3.2. Clinical Characteristics of Hypersensitivity to Titanium

Researchers have described various clinical manifestations in patients with allergies to titanium including episodes of hives, eczema, edema, reddening, and itching of the skin or mucosa, which may be localized, or generalized. They have also been associated with more serious problems such as atopic dermatitis, disturbed fracture healing, pain, necrosis, and weakening of orthopedic implants. In the field of implant dentistry, clinical manifestations include the appearance of facial erythema, disseminated facial eczema, contact dermatosis, atopic eczema, bullous eruptions, and proliferative hyperplasia tissue, edematous tissue, or non-keratinized tissue [1,14,19,21,45,46]. These various clinical manifestations of hypersensitivity to titanium are summarized in Table 3.

The orofacial region is associated with Type I, III, and IV allergic reactions. Type I is considered an immediate allergic reaction to external allergens with local and systemic anaphylaxis. In Type III reaction, a large quantity of circulatory antibodies is observed, produced between 2 and 8 h after implant placement, although they may appear after 14 days [1]. Type IV is considered the most frequently occurring allergy to metal, characterized by the local presence of abundant macrophages and T lymphocytes and the absence of B lymphocytes. Type IV, or delayed type, develops after repeated contact between an allergen and the skin or mucosa; it occurs during the first 24–72 h although the symptoms may appear at any time up to 14 days after surgery. Immune sensitivity may manifest at some distance from the implant and may even demonstrate a systemic reaction that remains unnoticed or may be incorrectly interpreted [1,13,20,37,47].

### 3.3. Tests for Identifying Metal Allergies

Various diagnostic tests are available to assess allergies to metals in general and titanium in particular. In epicutaneous patch testing (in vivo), substances located on the back or forearm are evaluated over a 3–7-day period [27,48]. The epicutaneous patch test is one of the most common and important tests for metal allergy.

Patch test reactions are interpreted by using criteria similar to International Contact Dermatitis Research Group (ICDRG) criteria: negative reaction, doubtful reaction (erythema only, no infiltration), weak positive reaction (erythema, infiltration, possibly discrete papules), strong positive reaction (erythema, infiltration, vesicles, papules), extreme positive reaction (erythema, infiltration, confluent vesicles), and irritant reaction [49].

However, because of the skin’s qualities of sealing and protection against direct contact, the test is not very sensitive, may give a false positive or negative, and only detects some 75% of Type IV metal allergies [21]. Lack of standardization may limit the use of a patch test. Nevertheless, it is the most widely used test despite the fact that it is not completely accepted to be the most effective [2,16,46,50].

In the cutaneous injection test (in vivo), the allergen is injected intradermally in the forearm. Red papules and vesicles are considered to show a positive result. This test is only recommended for Type I allergies and not for oral allergies [11,21]. The lymphocyte transformation test (LTT) is applied in vitro for mucosa-sensitizing allergens. Both local and systemic effects can be analyzed with this test [14,21]. The MELISA test (Memory Lymphocyte Immuno-Stimulation Assay) is a modification of the LTT (in vitro), which analyzes both local and systemic effects [3,18].

Muller et al. [19] compared its efficacy with the patch test, concluding that MELISA is more specific than the latter. Following the same line of research, Valentine-Thon et al. [51] concluded that MELISA is a useful tool for identifying and monitoring sensitivity in individuals exposed to metals. However, a study of the sparse scientific literature reveals that, as a consequence of its high number of false-positive results, this test is of no use for diagnosing metal allergy [52].

In this sense, Cederbrant et al. [53] compared the results of the patch test with those of the MELISA test, and those of a conventional LTT. The authors concluded that the MELISA test was useless because of its high number of false-positive results and that a positive patch test is considered to be the best indication for the presence of metal allergy (gold standard) [53,54].

Tests for metal allergies are summarized in Table 4.

### 3.4. Cases of Allergies to Titanium Dental Implants

The literature includes few articles that report cases of allergic hypersensitivity to titanium dental implants, and those that do exist describe isolated clinical cases (see Table 1).

Egusa et al. [14] report the case of a 50-year-old Japanese woman who presented disseminated facial eczema for 2 years, which appeared 1 week after mandibular dental implant placement. The Lymphocyte Transformation Test (LTT) indicated a reaction to titanium. Although the eczema worsened immediately after removing the implant, 6 months later it disappeared completely.

Hosoki et al. [16] published a case report of a 69-year-old Japanese man without any antecedents of allergy who received two dental implants in the right mandible in 2008. Two years later, he fractured his leg and was treated by means of titanium plates. Six months later, nummular eczema appeared. He was treated with antihistamines without any apparent improvement; the patch test was applied, which discovered allergies to tin, cobalt, palladium, indium, iridium, titanium, and copper. In 2011, having consolidated the leg fracture, the titanium plates were removed, and the eczema decreased by 50%. Later, other oral prostheses were removed, and the eczema decreased to 30%. Finally, after removing the dental implants, the eczema disappeared immediately.

Du Preez et al. [17] reported the case of a 49-year-old woman who had a steel plate implanted to consolidate a fractured metatarsus. She then received six Grade IV titanium inter-mentonian dental implants and was administered appropriate antibiotics and anti-inflammatories. A week later, slight swelling of the peri-implant soft tissues was observed, and so 400 mg metronidazole was prescribed. Five days later, the patient presented swelling in the submental region and labial sulcus, sharp pain, and hyperemia of the surrounding soft tissue. Both orthopantomography and occlusal radiography showed radiolucent lesions. In response to this sudden worsening it was decided to remove the implants, curetting the area. All the symptoms improved. Histological analysis of biopsies found foci of subacute inflammation and moderate chronic inflammation with lymphocytes, plasmatic cells, and histiocytes with concomitant fibrosis, as well as granulation tissue with isolated foreign body giant cells. It was concluded that the patient presented a Type IV allergic reaction, and it was suspected that the patient had been pre-sensitized as a result of the metatarsus steel plate. 

A transversal study by Hosoki et al. [18] analyzed allergies in 270 patients attending a Dental Metal Allergy Clinic with suspected metal allergies. Using the patch test, it was found that 217 patients showed positive for at least one metal allergen. Of 16 patients who had come to the clinic with allergy symptoms following titanium dental implant placement, five of them did not present positive test results for any metal, while the other 11 did, although only four presented allergies to titanium. In one of these four patients, all metal was removed from the oral cavity, including the implants, which resulted in noticeable improvements. In two of the patients, all metal was removed with the exception of the implants; these subjects also showed considerable improvement. The remaining patient of the four decided not to undergo removal of metals from the mouth but to receive treatment of the symptoms; this patient presented slight improvement. 

Müller and Valentine-Thon [19] analyzed 56 patients who developed clinical symptoms after receiving either titanium dental implants or titanium-based endoprostheses. These patients presented various symptoms including muscular or joint pain, nervous disorders, chronic fatigue syndrome, neurological problems, depression, multiple chemical sensitivity, dermatitis, and facial inflammation. The 56 patients received the Memory Lymphocyte Immunostimulation Assay (MELISA) test for titanium. Twenty-one showed positive, 16 uncertain, and 19 showed negative results. After removing the titanium structures/implants, the 54 patients who underwent this treatment presented remarkable clinical improvement.

Sicilia et al. [11] investigated allergies to titanium dental implants. Thirty-five patients out of a group of 1500 patients who underwent implant placement and then presented symptoms that suggested possible allergic reactions received the cutaneous injection test for titanium allergy. Of these 35 patients, 18 presented a positive reaction to titanium, while 35 randomly selected control subjects showed negative responses. Five out of eight inexplicable implant failures may have been attributable to titanium allergy, as these patients presented positive reactions in the test.

Finally, Mitchell et al. [20] presented two cases in their study in which both patients developed gingival hyperplasia after mandibular vestibuloplasty and the placement of a partial thickness skin graft after 2 weeks and 3.5 months of implant insertion, respectively. In both circumstances, traditional gingivectomy procedures, chemotherapeutic agents, and aggressive oral hygiene measures failed to adequately control the hyperplastic response. After replacing the titanium abutments with custom fabricated gold abutments, the epithelial condition seemed to return to normal.

### 3.5. Titanium Allergy Management

The scientific literature includes very few indications or instructions for the management of patients undergoing a process of hypersensitivity to a titanium dental implant. As already mentioned, this is because dental professionals consider that titanium one of the most biocompatible metals for implantation in the body; they do not believe that allergic reactions will appear or that they have sufficient clinical relevance to warrant an established protocol for dealing with them.

As stated earlier, the allergic reaction to titanium can follow the presence of ions derived from the corrosion of the dental implant. Consequently, guidelines should be drawn up before starting treatment; if and when a hypersensitivity reaction following titanium dental implant placement occurs, a range of treatment alternatives should be clearly established and made available.

## 4. Discussion

The scientific literature repeatedly insists on the success of titanium implants. Nevertheless, the so-called tribocorrosion process releases titanium ions into the surrounding tissues, which can trigger a cascade of reactions, localized or at a distance, or even systemic reactions [1,14,19,21,35,45,46].

As demand grows for implant-based dental treatments, the range of implants available on the market is expanding exponentially. When it comes to product development, the choice of implant material, surface treatment, and cleaning technique are key factors that will characterize the finished product. However, competition between brands is tough, a situation that sadly sometimes places business interests and cost-cutting before quality control. Thus, Zinelis et al. [55] showed greater carbon contamination, possibly related to cleaning procedures after surface treatments, which affects the protein and cell adsorption phenomena [56]. Likewise, Massaro et al. [57] mention that the presence of Na, K, and Cl contaminants, occasionally detected by X-ray Photoelectron Spectroscopy (XPS) and High Vacuum X-ray Energy Dispersive Microanalysis (HV-EDX) on implant surfaces, may be residues of proprietary cleaning treatments performed after sandblasting. The biological role of these contaminants remains unclear.

Various tests for allergy to titanium are described in the literature, the patch test being the most frequently used [2,16,46,50].

As far as the authors are aware, this scoping review represents one of the first attempts to summarize the current, relevant knowledge about allergies to titanium dental implants and their management. The scientific literature on this topic it is very scarce, which highlights the need to establish a protocol for those patients who are sensitized to titanium before and after the placement of dental implants. In patients with a history of metal allergies, it is advisable to perform a titanium allergy test. The alloy and its purity must be identified together with any trace elements it may contain in order to optimize diagnosis [40,58,59].

When dental implants are placed in the jaws, and then the patient proves allergic to titanium, the option of explanation must be considered, basing the decision on the ratio of risk/benefit to the patient. Having removed titanium implants, if the patient shows no clinical improvement, the option of removing all metal prostheses from the organism should be considered. In this context, zirconium dioxide implants (ZrO2) or yttria-stabilized zirconia offer a promising alternative to titanium as no case of allergy to zirconium oxide has ever been reported [60,61,62,63,64,65]. Nevertheless, in animal experiments zirconia implants have been shown to release particles but only (approximately) half the quantity released by titanium implants [66].

## 5. Conclusions

Within the limitations of this literature review, it may be concluded that allergies to titanium are uncommon, although they do present a real possibility that should not be overlooked when it comes to treating patients requiring prosthodontic rehabilitation with dental implants, as an allergy can trigger a range of symptoms. The literature points to a lack of standardization in research into titanium allergy. Further studies are needed with adequate protocols, sample sizes, and follow-up periods, which would obtain clearer and more reliable results.

## Figures and Tables

**Table 1:** Case Reports and Clinical Studies related to allergies to titanium dental implants.

Authors and Year	Study	Number of Patients (Mean Age/Range in Years)	Gender	Number of Implants	Subjects with Ti Allergy Compared with the Overall Sample	Time Lag between Placement of Implants and Subsequent Explantation	Clinical Signs of Associated Allergy	Conclusions
Sicilia et al. 2008 [11]	Clinical/Retrospective	35 (50.2/21–68)	10 M 25 F	NA	9/1500	NA	Rash, urticaria, pruritus, redness, dermatitis and facial eczema	Titanium allergy can be detected in dental implant patients
Egusa et al. 2008 [14]	Case-Report	1 (50-year-old)	F	2		2 years	Facial eczema	Allergic reactions can be detected in patients with Ti dental implants.
Hosoki et al. 2016 [16]	Case-Report	1 (69-year-old)	F	2		6 years	Nummular eczema	Pre-implant patients should be asked about a history of hypersensitivity reactions to metals and patch testing should be recommended.
du Preez et al. 2007 [17]	Case-Report	1 (49-year-old)	F	6		1 week	Pain, hyperaemia of soft tissues, swelling in submental and labial sulcus	A chronic inflammatory response with fibrosis was observed around all Ti implants, which could indicate a real allergy to titanium dental implants
Hosoki et al. 2018 [18]	Clinical Retrospective	270 (53, 9/7–85) 16 with titanium dental implants	7 M, 9 F	NA	4/16	NA	Soreness, rash, itching, urticaria, discomfort, stomatitis, lichen planus, pustulosis palmaris et plantaris, crazing of a nail	Titanium allergy in patients caused by dental implants exists
Müller et al. 2006 [19]	Clinical and Experimental	56 (53.8/14.3–84.1)	17 M 39 F	NA	21/56	6 months	Dermatitis and acne-like facial inflammation	Ti can induce clinically relevant hypersensitivity and other immune dysfunctions in certain patients chronically exposed to this reactive metal.
Mitchell et al. 1990 [20]	Case-report	1 (49-year-old)	F	4	1	2 weeks	Gingival hyperplasia	Hyperplasia in the gingival tissues may occur in patients with Ti dental implants.
		1 (44-year-old)	M	4	1	3,5 months	Gingival hyperplasia	

**Table 2:** In-vivo experiments.

Article	Animal and Number	Administration	Conclusions
Olmedo et al. 2008 [31]	Rats (*n* = 20)	Titanium dioxide (TiO2) preparation injection	Internal exposure will lead to a concentration of titanium ions in the surrounding tissues, lymph nodes, and even in lung or liver tissue
Olmedo et al. 2011 [32]	Rats (*n* = 62)	Titanium dioxide (TiO2) preparation injection	Biokinetics will be influenced by the biomechanical properties of titanium particles
Frisken KW et al. 2002 [33]	Sheep (*n* = 12)	Titanium Dental Implants	The presence of titanium found in the lungs in cases of failed implants was 2.2–3.8 times higher than when implants were successful, while in local lymph nodes, it was 7.0–9.4 times higher. Ambiguous results were found in the liver and spleen.

**Table 3:** Clinical manifestations of hypersensitivity to titanium.

Localization	Symptoms
Local Manifestations	Hives, edema, eczema, reddening, and itching of the skin or mucosa, erythema, contact dermatosis, atopic eczema, bullous eruptions, proliferative hyperplasia tissue/edematous tissue /non-keratinized tissue, peripheral giant cell pyogenic granuloma
Manifestations at a distance from the implant place	Hives, disseminated facial eczema, edema, reddening, and itching of the skin or mucosa, atopic dermatitis
Systemic reactions	Pain, necrosis, weakening of orthopedic implants, disturbed fracture healing, nervous disorders, chronic fatigue syndrome, neurological problems, depression, multiple chemical sensitivity

Siddiqui et al. [1], Egusa et al. [14], Müller et al. [19], Chaturvedi et al. [21], Javed et al. [45], Olmedo et al. [46].

**Table 4:** Tests for metal allergies.

Test	In vivo/In vitro	Application Site	Evaluation Time	Positive Signs	Comments
Epicutaneous Patch Test [2,3,11,16,18,19,21,27,48,49,50,51,53,54]	In vivo	Titanium dioxide (TiO2) preparation patch on back or forearm	3–7-day period	-Negative reaction -Doubtful reaction (erythema only, no infiltration) -Weak positive reaction (erythema, infiltration, possibly discrete papules) -Strong positive reaction (erythema, infiltration, vesicles, papules) -Extreme positive reaction (erythema, infiltration, confluent vesicles) -Irritant reaction	-One of the most common and important tests for metal allergy (Gold Standard). -The test is not very sensitive, may give a false positive or negative, only detects 75% of Type IV metal allergies. -Lack of standardization may limit their use
Cutaneous Injection Test [11,21,27,49]	In vivo	Titanium dioxide (TiO2) preparation injection in forearm	15–30 min	Red, papular, and/or vesicular reaction of the skin is considered as positive	-Only recommended for Type I allergies and not for oral allergies
Lymphocyte Transformation Test (LTT) [3,14,21,27,53,54]	In vitro	In vitro, heparinized venous blood	5 days	Stimulation index ≥2.0	-Analyzes local and systemic effects
Memory Lymphocyte Immuno-Stimulation Assay (MELISA) [3,19,21,27,51,52,53,54]	In vitro	In vitro, defibrinated blood	5 days	Stimulation index ≥3.0 together with the presence of lymphoblasts	-Analyzes local and systemic effects

de Graaf et al. [2], Wood et al. [3], Sicilia et al. [11], Egusa et al. [14], Hosoki et al. [16], Hosoki et al. [18], Müller et al. [19], Chaturvedi et al. [21], Vijayaraghavan et al. [27], Kittagawa et al. [48], Fregert et al. [49], Dennis et al. [50], Valentine-Thon et al. [51], Koene et al. [52], Cederbrandt et al. [53], Cederbrandt et al. [54].
